# Hemoglobin induced NO/cGMP suppression Deteriorate Microcirculation via Pericyte Phenotype Transformation after Subarachnoid Hemorrhage in Rats

**DOI:** 10.1038/srep22070

**Published:** 2016-02-25

**Authors:** Qiang Li, Yujie Chen, Bo Li, Chunxia Luo, Shilun Zuo, Xin Liu, John H. Zhang, Huaizhen Ruan, Hua Feng

**Affiliations:** 1 Department of Neurosurgery, Southwest Hospital, Third Military Medical University, Chongqing, China; 2 Department of Neurobiology, College of Basic Medical Sciences, Third Military Medical University, Chongqing, China; 3 Department of Neurosurgery, Jinan Military General Hospital, Jinan, Shandong, China; 4 Department of Neurology, Southwest Hospital, Third Military Medical University, Chongqing, China; 5 Department of Physiology and Pharmacology, Loma Linda University, Loma Linda, California, USA

## Abstract

Subarachnoid hemorrhage (SAH) usually results from ruptured aneurysm, but how leaked hemoglobin regulates the microcirculation in the pathophysiology of early brain injury after SAH is still unclear. In the present study, we sought to investigate the role and possible mechanism of hemoglobin induced pericyte phenotype transformation in the regulation of microcirculation after SAH. Endovascular perforation SAH rat model, brain slices and cultured pericytes were used, and intervened with endothelial nitric oxide synthase (eNOS) antagonist L-NNA and its agonist scutellarin, hemoglobin, DETA/NO (nitric oxide(NO) donor), PITO (NO scavenger), 8-Br-cGMP (cGMP analog). We found modulating eNOS regulated pericyte α-SMA phenotype transformation, microcirculation, and neurological function in SAH rats. Modulating eNOS also affected eNOS expression, eNOS activity and NO availability after SAH. In addition, we showed hemoglobins penetrated into brain parenchyma after SAH. And hemoglobins significantly reduced the microvessel diameters at pericyte sites, due to the effects of hemoglobin inducing α-SMA expressions in cultured pericytes and brain slices via inhibiting NO/cGMP pathway. In conclusion, pericyte α-SMA phenotype mediates acute microvessel constriction after SAH possibly by hemoglobin suppressing NO/cGMP signaling pathway. Therefore, by targeting the eNOS and pericyte α-SMA phenotype, our present data may shed new light on the management of SAH patients.

Despite years of research, early brain injury, which mainly contributes to the mortality and poor prognosis after subarachnoid hemorrhage (SAH), remains a feared complication of ruptured intracranial aneurysm^1^. It usually occurs within the first 3 days after initial bleed with severe reduction in cerebral blood flow and subsequent ischemic brain damage^2^, while large vessel spasm usually starts at 5 to 8 days after SAH^3^. Therefore, the harmony of microcirculation might be the promising therapeutic target to improve clinical outcomes of patients with SAH^4^,^5^.

Dysfunction of endothelial nitric oxide synthase (eNOS) had been demonstrated to involve the microvascular related damages after SAH^6^, but the detailed mechanism remains unclear. Pericyte, the microvessel contraction handler, was recently considered as the main participant of microcirculation regulation in SAH pathophysiology^7^,^8^,^9^. Under several pathological conditions, pericyte was induced to transform into alpha-smooth muscle actin (α-SMA) positive phenotype, exhibit contractile function and secret cytokine, including matrix metalloproteinase 9 and matrix metalloproteinase 2, to regulate the endothelial barrier integrity^10^,^11^,^12^.

The eNOS product, nitric oxide (NO), has high affinity for hemoglobin to act as an endothelial derived relaxing factor, which has been accepted as a cause of vasospasm^13^,^14^. However, it is still unclear how hemoglobin impacts the microcirculation after SAH. Thus, the present study sought to investigate the role and possible mechanism of pericyte phenotype transformation in the regulation of microcirculation after subarachnoid hemorrhage.

## Results

### Modulating eNOS affected eNOS expression, eNOS activity and NO availability after SAH

**Figure 1:**
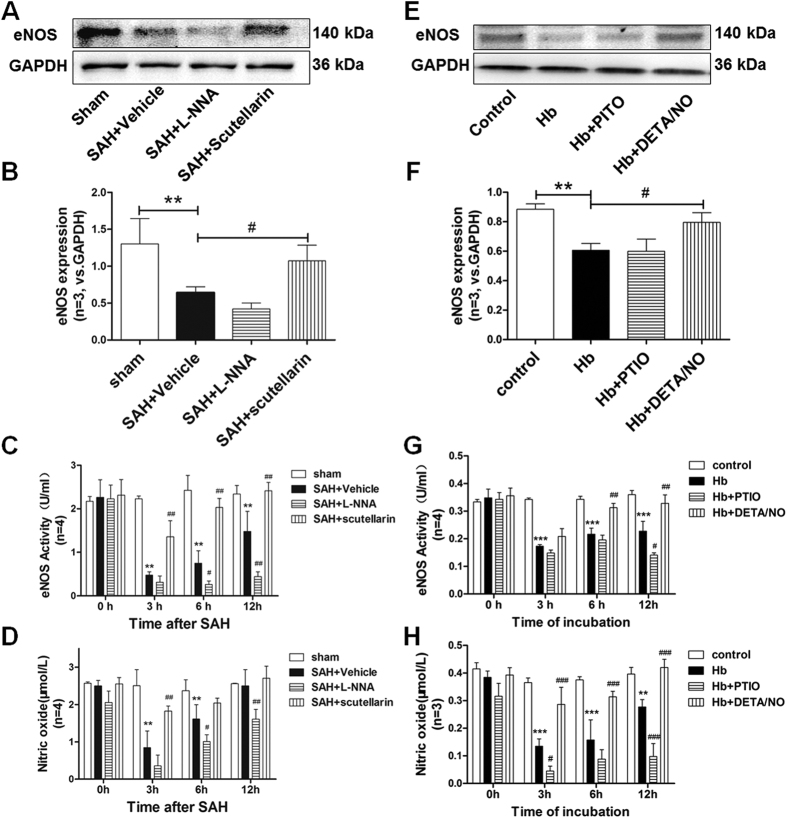
Effects of modulating eNOS on eNOS expression, eNOS activity, and nitric oxide content after SAH. (**A**) Representative western blot bands and (**B**) quantification analysis of eNOS expressions at ipsilateral cortex in each group (n = 3). The cropped bands had been run under the same experimental conditions. (**C**) Quantification analysis of eNOS activity at ipsilateral cortex in each group (n = 4). (**D**) Quantification analysis of nitric oxide content at ipsilateral cortex in each group (n = 4). (**E**) Representative western blot bands and (**F**) quantification analysis of eNOS expressions in cultured pericytes of each group (n = 3). The cropped bands had been run under the same experimental conditions. (**G**) Quantification analysis of eNOS activity in cultured pericytes of each group (n = 4). (**H**) Quantification analysis of nitric oxide content in cultured pericytes of each group (n = 3) eNOS: endothelial nitric oxide synthase; All data were presented as mean ± standard deviation. Data were analyzed by one-way analysis of variance followed by Newman-Keuls multiple comparison method. *p < 0.05 vs. sham; **p < 0.01 vs. sham; ^#^p < 0.05 vs. SAH + Vehicle; ^##^p < 0.01 vs. SAH + Vehicle.

To evaluate the effects of modulating eNOS, we examined the eNOS expression, eNOS activity, and NO availability by western blot and relative assay kit. At 3 hours after SAH, the protein level of eNOS was significantly decreased comparing with the sham group (p = 0.0300). L-NNA treatment showed no statistical effects on the eNOS expression after SAH (p = 1.0000), while scutellarin significantly increased the protein level of eNOS comparing with SAH rats (p = 0.0291) (Fig. 1A,B). Compared to sham rats, eNOS activity and NO availability was significantly decreased at 3 and 6 hours after SAH (p = 0.0010), but only eNOS activity decreased at 12 hours after SAH (p = 0.0001) while NO availability exhibited no significant difference at 12 hours after SAH (p = 0.9623). Compared to SAH group, L-NNA decreased eNOS activity and NO availability at 6 and 12 hours after SAH (p = 0.0044), whereas scutellarin treatment significantly increased eNOS activity and NO availability in SAH rats (p = 0.0001) except at 12 hours after SAH (p = 0.3680) (Fig. 1C,D). In addition, we also evaluated the eNOS expression, eNOS activity and NO availability in cultured pericyte, and found the same trend as *in vivo* study(Fig. 1E–H). These data indicated that the decreasing of eNOS and NO after SAH.

### Modulating eNOS regulated microcirculation and neurological function after SAH

To clarify the effects of modulating eNOS on microcirculation and neurological deficits after SAH, we employed the modified Garcia scale and *in vivo* microvessels observation.

Comparisons of SAH grading score revealed no significant differences among the groups at 24 hours after SAH (p = 0.8063, Supplemental Fig. 1B). None of the sham-operated rats died, and 17 rats (6 in SAH + Vehicle group, 6 in SAH + L-NNA group, and 5 in SAH + scutellarin group) died within 24 hours after SAH due to severe hemorrhagic volume. No other adverse events were observed in the rest of the rats.

**Figure 2:**
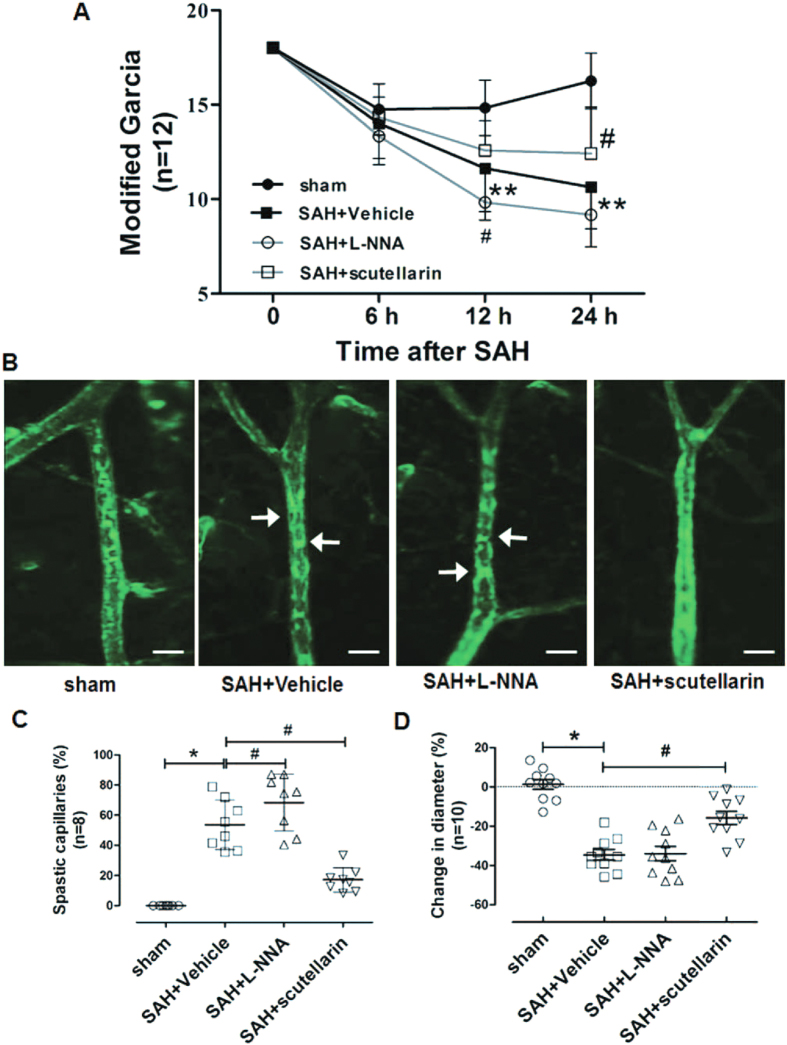
Effects of modulating eNOS on neurological functions and microcirculation after SAH (**A**) Modified Garcia scores of each group at 6, 12, and 24 hours after SAH. (**B**) Representative imaging of microvessels affiliated to middle cerebral artery by intravital fluorescence microscopy at the ipsilateral cortex in each group at 3 hours after SAH (n = 12). (**C**) Quantification analysis of the percent of spastic microvessels affiliated to middle cerebral artery at the ipsilateral cortex (n = 8). (**D**) Quantification analysis of the diameter of microvessels affiliated to middle cerebral artery at the ipsilateral cortex (n = 12). All data were presented as mean ± standard deviation. Chi-square test was used for the statistical analysis of neurological deficits between different time and groups. For statistical analysis of microvessels, independent samples t-test was used. *p < 0.05 vs. sham; **p < 0.01 vs. sham; ^#^p < 0.05 vs. SAH + Vehicle.

Compared with sham group, SAH rats showed significant neurological deficits on the modified Garcia test at both 12 and 24 hours after SAH (p = 0.0030), but not at 6 hours after SAH (p = 0.6210). L-NNA treatment significantly aggravated neurological deficits at 12 hours after SAH by comparing with the SAH group (p = 0.0174). On the other hand, scutellarin markedly alleviated the neurological deficits at 24 hours after SAH (p = 0.0195) (Fig. 2A).

No microvessel constrictions were observed in sham-operated rats, but numerous discontinuous constricted microvessels were observed at 3 hours after SAH in SAH + Vehicle and SAH + L-NNA groups (Fig. 2B). Interestingly, the pattern is similar to the pearl string-like constrictions observed in SAH patients. And those phenomena were alleviated after scutellarin treatment (Fig. 2B). Quantification of these observations was exhibited by the percentage of spastic microvessels (Fig. 2C). At 3 hours after SAH, 54.2% of counted middle cerebral artery branches showed one or more constrictions comparing with sham rats (p = 0.0001), and microvessel constrictions were significantly increased (68.2% of counted capillaries) in SAH + L-NNA group (p = 0.0422). However, only 17.1% of counted capillaries were affected in the SAH + scutellarin group (p = 0.0001 vs. SAH + Vehicle group). In addition, the severity of microvessel constriction was shown by the changes of microvessel diameter (Fig. 2D). At 3 hours after SAH, affected vessel segments were constricted by an average of 35 ± 8% comparing with sham group (p = 0.0001). In the SAH + L-NNA group, the average constriction was 34 ± 11% comparing with SAH rats (p = 1.0000). Scutellarin treatment, by contrast, significantly alleviated the constriction with an average of 16 ± 10% (p = 0.0010).

These data exhibited the microvessel constriction and following neurological deficits after SAH, which could be alleviated by eNOS agonist.

### Modulating eNOS affected the expression of α-SMA after SAH

**Figure 3:**
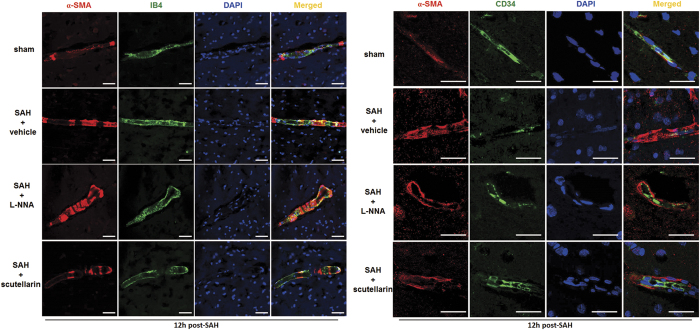
Effects of modulating eNOS on α-SMA expression after SAH Representative pictures of immunofluorescence staining for α-SMA expressions at 12 hours after SAH. Scale Bar = 20 μm; n = 3 in each group.

**Figure 4:**
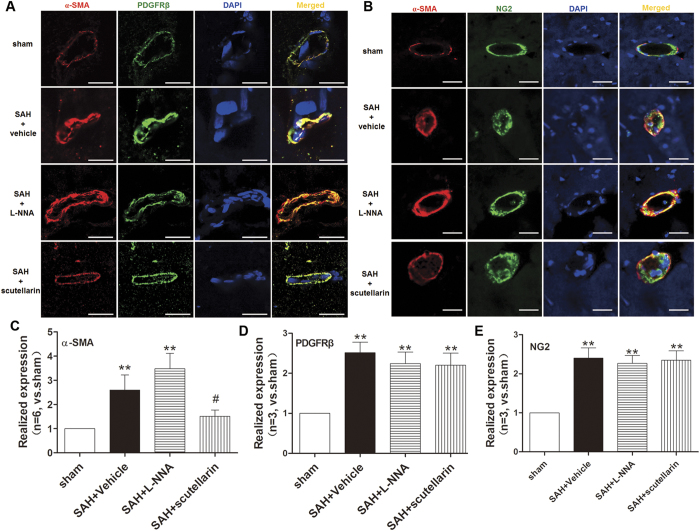
The α-SMA expressions of pericytes after SAH. (**A**) Representative double immunofluorescence pictures of the co-localization of PDGFRβ and α-SMA at 12 hours after SAH. (**B**) Representative double immunofluorescence pictures of the co-localization of NG2 and α-SMA at 12 hours after SAH. And quantification analysis of the fluorescence intensity in (**C**) α-SMA, (**D**) PDGFRβ and (**E**) NG2 in each group (n = 6 for α-SMA, n = 3 for PDGFRβ and NG2). PDGFRβ: platelet-derived growth factor receptor beta; Scale Bar = 20 μm; n = 3 in each group. All data were presented as mean ± standard deviation. Data were analyzed by one-way analysis of variance followed by Newman-Keuls multiple comparison method. *p < 0.05 vs. sham; **p < 0.01 vs. sham; ^#^p < 0.05 vs. SAH + Vehicle; ^##^p < 0.01 vs. SAH + Vehicle.

To investigate the possible mechanism of the dilation effects of eNOS, we examined the localization and expression of α-SMA. Immunofluorescence staining showed the expression of α-SMA was enhanced at 12 hours after SAH. And much stronger expression of α-SMA was observed after L-NNA treatment. However, scutellarin treatment down-regulated the expression of α-SMA after SAH (Figs 3 and 4A–C).

In addition, double immunofluorescence labeling showed platelet-derived growth factor receptor beta (PDGFRβ) was co-localized with CD34 and α-SMA in capillaries, respectively (Fig. 4A, Supplemental Fig. 2). However, the expression of PDGFR-β only increased in SAH + vehicle group (vs. Sham group), but slightly decreased after L-NNA or scutellarin treatment with no statistical differences (vs. SAH + vehicle group). And NG2 expression exhibited the same trend with PDGFR-β (Fig. 4A,B,D,E).

Our data suggested that α-SMA expressed on pericyte could be inhibited by eNOS activity, while the microvessel constriction also be alleviated.

### Distribution of leaked blood and hemoglobin after SAH

**Figure 5:**
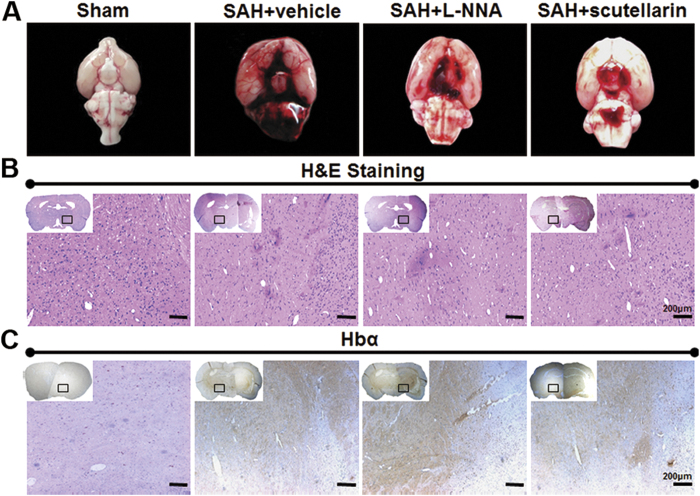
Distribution of leaked blood and hemoglobin at 12 hours after SAH. (**A**) Representative pictures of the skull base after SAH surgery. (**B**) Represetative pictures of hematoxylin and eosin staining for blood distribution after SAH. (**C**) Representative pictures of immunohistochemistry staining for hemoglobin-α. Hbα: hemoglobin-α; Scale bars = 200 μm; n = 3 in each group.

At 12 hours after SAH, extensive bleeding induced filament puncture was still obvious, which was particularly pronounced around the circle of Willis and along the ventral brainstem. Furthermore, leaked blood and hemoglobin were seen in the large areas of the brain parenchyma (Fig. 5).

### Hemoglobin incubation regulated the microvessel diameters at pericyte sites in brain slices

After SAH, the fresh blood leaked out of the cerebral arties and distributed irregularly around and into the parenchyma. To explore the potential role of hemoglobin in regulating microcirculation, immunohistochemistry staining of brain tissues and *in vitro* brain slices were used in section.

**Figure 6:**
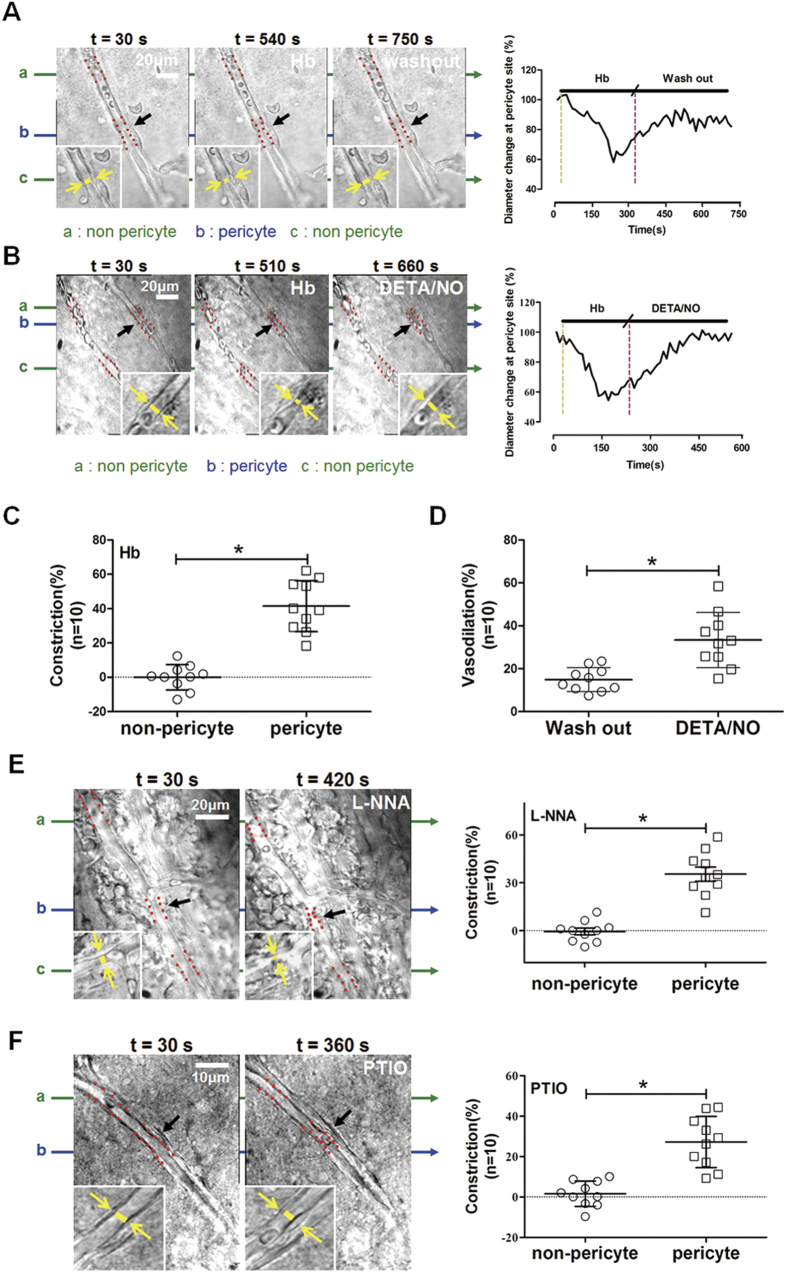
Effects of hemoglobin and eNOS/nitric oxide signal on microvessel constriction of *in vitro* brain slices. (**A**) Microvessel in cortical slice successively exposed to artificial cerebrospinal fluid, hemoglobin and artificial cerebrospinal fluid. (**B**) Microvessel successively exposed to artificial cerebrospinal fluid, hemoglobin and DETA/NO. (**C**) Microvessel constriction evoked by hemoglobin at pericyte sites and non-pericyte sites. (**D**) Better alleviative effect was observed in DETA/NO compared to washout only. (**E**) Left: Inhibition of eNOS by L-NNA evoked microvessel constriction; Right: Microvessel constriction at pericyte and non-pericyte sites. (**F**) Left: Depletion of nitric oxide by PTIO evokes microvessel constriction. Right: Microvessel constriction at pericyte and non-pericyte sites. Black arrow represents pericyte; green arrow represents microvessel with non-pericyte; blue arrow represents microvessel with pericyte; and red plots indicate the microvessel wall. All data were presented as mean ± standard deviation. For statistical analysis of microvessels, independent samples t-test was used.*p < 0.05 vs. non-pericyte or washout. n = 10 in each group.

To directly assess the effect of eNOS derived NO on microvessel constriction, a slices model was treated with hemoglobin, DETA/NO, L-NNA and PTIO, respectively. Hemoglobin evoked microvessel constriction at pericyte sites, but not at non-pericyte sites (p = 0.0497, Fig. 6A,C), and the constriction was alleviated by washout (Fig. 6A) and DETA/NO (Fig. 6B). Further analysis illustrated that DETA/NO exhibited significantly stronger vasodilation effect than washout (p = 0.0197, Fig. 6D). On the contrary, either L-NNA or PTIO triggered microvessel constriction at pericyte sites (p = 0.0333, Fig. 6E,F). These data indicated hemoglobin could reduce the microvessel diameters at pericyte sites.

Hemoglobin reduced the protein levels of eNOS in a time frame of a few hours, suggesting that it might suppress eNOS expression or accelerate its degradation. However, *in vitro* experiments of brain slices with hemoglobin or eNOS inhibitor found that microvessel constriction was induced in 30–750 seconds with maximal effect in 150–200 seconds. We understand the time frame differences between *in vivo* and *in vitro* studies. To address this question, we did the time line of hemoglobin incubation on NO availability and eNOS expression. The data showed that after 10 minutes’ hemoglobin incubation in cultured brain slices, the NO content significantly decreased comparing to control incubation (p = 0.0038, Supplemental Fig. 3A). But the eNOS expression showed no statistical difference at the same time (p = 0.2053, Supplemental Fig. 3B), and significantly decreased after 30 minutes’ hemoglobin incubation (p = 0.0487, Supplemental Fig. 3B). These results indicated that hemoglobin may cause microvessel constriction through NO scavenging in the very short time^15^,^16^, while reduce eNOS expression later to sustain the effect^17^.

### Hemoglobin induced the expression of α-SMA via NO/cGMP pathway in cultured pericytes and brain slices

To explain the potential involvement of NO/cGMP signaling pathway in pericyte phenotype transformation, cultured pericytes and brain slices were conducted to hemoglobin incubation and NO/cGMP modulating.

**Figure 7:**
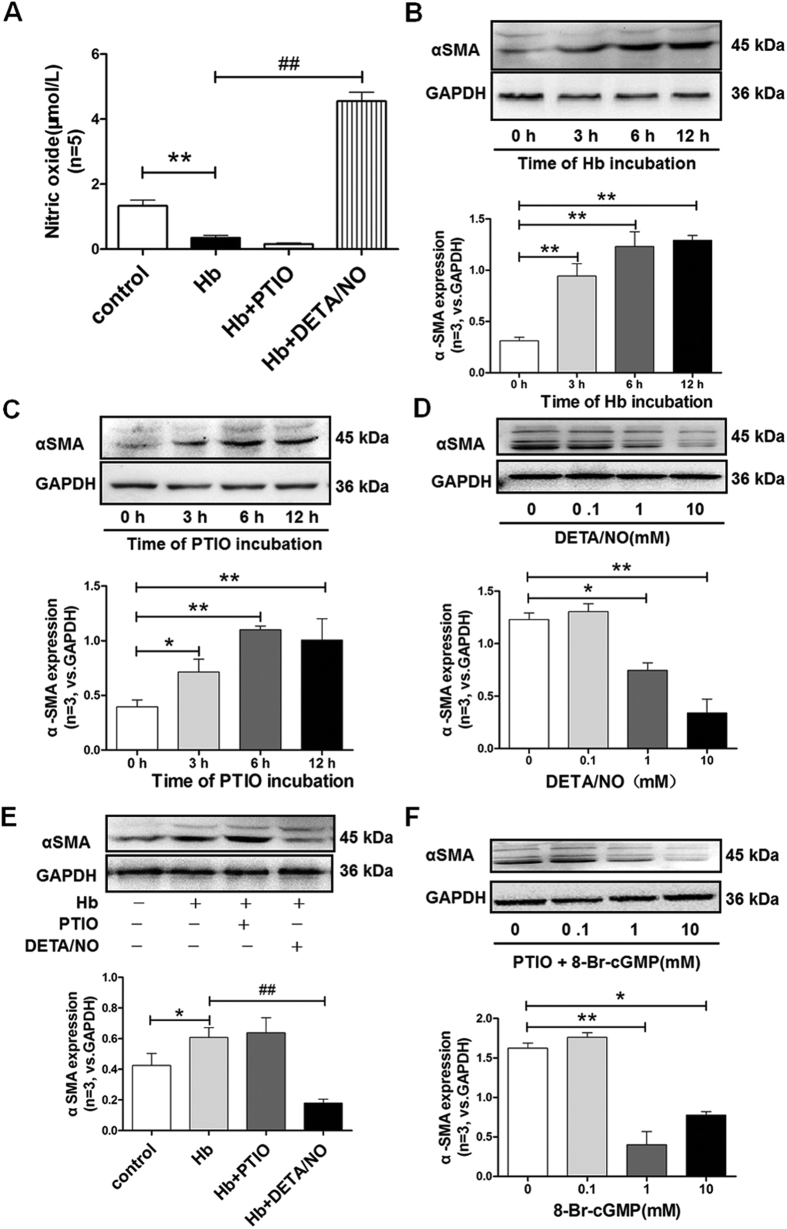
Effects of nitric oxide/cGMP signal on the protein levels of α-SMA in cultured pericytes. (**A**) Quantification analysis of nitric oxide content in culture bath after 3 hours incubation in each group. (**B**) Representative western blot bands and quantification analysis of α-SMA at 3, 6, and 12 hours after hemoglobin incubation. (**C**) Representative western blot bands and quantification analysis of α-SMA at 3, 6, and 12 hours after PTIO incubation. (**D**) Representative western blot bands and quantification analysis of α-SMA at 3 hours after DETA/NO incubation with gradient concentrations. (**E**) Representative western blot bands and quantification analysis of α-SMA after 3 hours’ incubation in each group. (**F**) Representative western blot bands and quantification analysis of α-SMA at 3 hours after 8-Br-cGMP incubation with gradient concentrations in PTIO pretreated pericytes. The cropped bands had been run under the same experimental conditions. All data were presented as mean ± standard deviation. Data were analyzed by one-way analysis of variance followed by Newman-Keuls multiple comparison method. *p < 0.05 vs. Control or zero hours after incubation; **p < 0.01 vs. Control or zero hours after incubation; ^##^p < 0.01 vs. Hemoglobin; n = 3 in each group.

Cultured pericytes in the present study exhibited abundant cytoplasm, triangular and polygonal cell morphology, and were positively stained by α-SMA and PDGFR-β (Supplemental Fig. 1C). Compared to the control group, hemoglobin reduced the concentration of NO at 3 hours after incubation in the culture bath (p = 0.0046, Fig. 7A), and increased the mRNA and protein levels of α-SMA at 3, 6, 12 hours after incubation in cultured pericytes (p = 0.0001, Fig. 7B, Supplemental Fig. 4A). And, PTIO incubation also increased the mRNA and protein levels of α-SMA at 3, 6, 12 hours after incubation in cultured pericytes (p = 0.0005, Fig. 7C, Supplemental Fig. 4B). On the other hand, DETA/NO incubation significantly decreased the pericyte α-SMA expression in a gradient concentration manner (p = 0.0015, Fig. 7D).

In addition, compared to hemoglobin solo incubation, DETA/NO significantly increased the NO availability and decreased the expression of α-SMA in hemoglobin incubated pericytes (p = 0.0002, Fig. 7A,E Supplemental Fig. 3 C). However, there were no significant differences of NO availability and α-SMA expression between the hemoglobin and hemoglobin + PTIO groups (p = 0.6033, Fig. 7A,E, Supplemental Fig. 4C). Furthermore, 8-Br-cGMP incubation significantly decreased the expression of α-SMA in PTIO pretreated pericytes, with the dosage between 1 to 10 mM (p = 0.0001, Fig. 7F).

**Figure 8:**
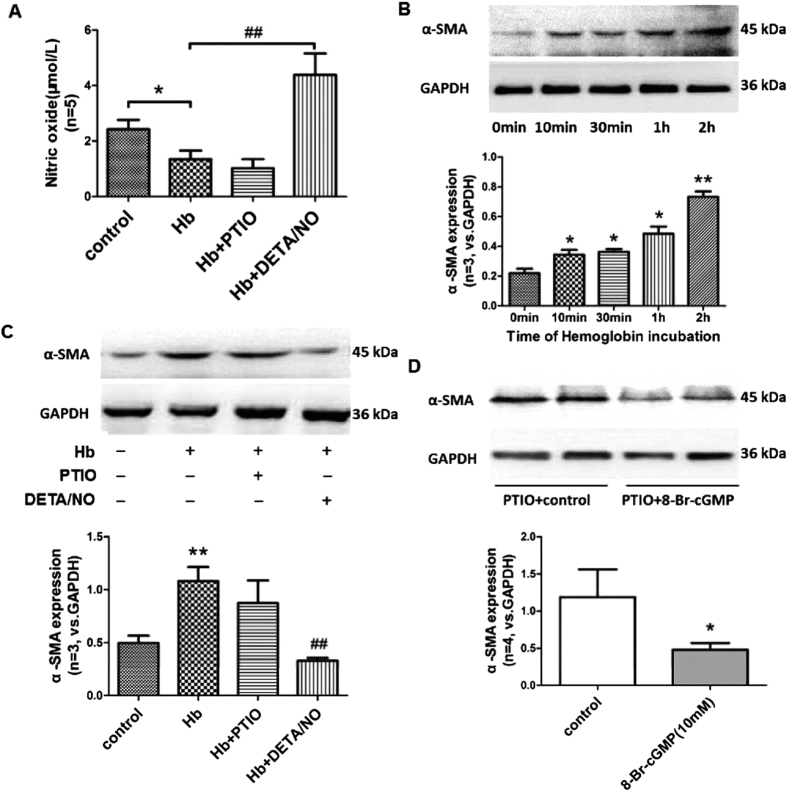
Effects of nitric oxide/cGMP signal on the protein levels of α-SMA in cultured brain slices. (**A**) Quantification analysis of nitric oxide content in culture brain slices after 3 hours’ incubation in each group. (**B**) Representative western blot bands and quantification analysis of α-SMA at 0, 10, 30 minutes and 1, 2 hours after hemoglobin incubation. (**C**) Representative western blot bands and quantification analysis of α-SMA after 3 hours’ incubation in each group. (**D**) Representative western blot bands and quantification analysis of α-SMA at 3 hours after PTIO pretreated brain slices with or without 8-Br-cGMP incubation. The cropped bands had been run under the same experimental conditions. All data were presented as mean ± standard deviation. Data were analyzed by one-way analysis of variance followed by Newman-Keuls multiple comparison method. *p < 0.05 vs. Control or zero minutes after incubation; **p < 0.01 vs. Control or zero hours after incubation; ^##^p < 0.01 vs. Hemoglobin; n = 5 for NO content, n = 4 for PTIO with or without 8-Br-cGMP incubation, n = 3 for others.

Similar with the results in cultured pericytes, our data indicated that hemoglobin reduced the concentration of NO at 2 hours after incubation in cultured brain slices (p = 0.0302, Fig. 8A), and increased the protein levels of α-SMA at 10 minutes, 30 minutes, 1 hours, 2 hours after incubation in cultured brain slices (p = 0.0474, 0.0236, 0.0155, 0.0033, respectively, Fig. 8B). On the other hand, compared to hemoglobin solo incubation, DETA/NO significantly increased the NO availability and decreased the expression of α-SMA in hemoglobin incubated brain slices (p = 0.0008, Fig. 8A,C). However, there were no significant differences of NO availability and α-SMA expression between the hemoglobin and hemoglobin + PTIO groups (p = 0.3229, 0.2086, respectively, Fig. 8A,C). Furthermore, 8-Br-cGMP incubation significantly decreased the expression of α-SMA in PTIO pretreated brain slices (p = 0.0451, Fig. 8D).

Combine the data from cultured pericytes and brain slices, the results demonstrated the involvement of NO/cGMP signal in the effects of hemoglobin on α-SMA.

## Discussion

In the present study, we investigated the role and possible mechanism of hemoglobin induced microcirculation dysfunction after subarachnoid hemorrhage, and found that the eNOS expression and activity, NO availability, cerebral microvessel diameters were significantly reduced to exhibit neurological deficits after SAH. eNOS antagonist L-NNA treatment aggravated those changes, while eNOS agonist scutellarin significantly increased the eNOS expression and activity, NO availability and cerebral microvessel diameters comparing to the SAH group. We also found that the pericyte marker PDGFRβ was co-localized with endothelium marker CD34 and α-SMA in microvessels, respectively. And the expression of α-SMA was increased by L-NNA, but decreased by scutellarin treatment. In addition, amount of hemoglobin was observed around cerebral microvessels in the large areas of the brain parenchyma. Thus, we used hemoglobin to incubate *in vitro* brain slices and found the microvessel diameters were decreased at pericyte sites, but not at non-pericyte sites. And the constriction was alleviated by washout and a nitric oxide donor DETA/NO, whereas L-NNA and nitric oxide scavenger PITO also evoked microvessel constriction. Furthermore, hemoglobin incubation significantly decreased the NO concentration and increased α-SMA expression in cultured pericytes and brain slices. PTIO exhibited similar effects on the expression of α-SMA, while DETA/NO decreased α-SMA expression in cultured pericytes and brain slices. Further study showed that DETA/NO significantly increased the NO availability and decreased the expression of α-SMA in hemoglobin incubated pericytes and brain slices. And cGMP analog 8-Br-cGMP incubation significantly decreased the expression of α-SMA in PTIO pretreated pericytes and brain slices.

SAH patients experience cerebral ischemia in the first few days after hemorrhage, even though large cerebral vessels do not show any signs of vasospasm^18^. Thus, the perfusion deficit has to be attributed to changes at the level of the cerebral microcirculation—as already pointed out by Bederson *et al.*^19^. This hypothesis was subsequently demonstrated by a study performed in SAH patients during aneurysm clipping within the first 3 days after the initial bleed^20^, in which the subarachnoid microvessels showed pearl string like contractions, indicating acute microvessel constriction plays a critical role in the pathophysilogy of early brain injury after SAH^21^.

Pericytes wrap around endothelial cells and play critical roles in supporting the growth and maintenance of blood vessels^7^,^22^. Previous work had indicated that pericytes bidirectionally control the microvessel diameter and regulate the cerebral blood flow^23^. And pericyte α-SMA phenotype exhibited contractile function and secret cytokines to regulate the endothelial barrier integrity^10^,^11^,^12^. In the present study, we observed the existence of pericyte α-SMA phenotype transformation caused acute microvessel constriction and neurological deficits in the setting of SAH. These findings demonstrated that pericytes were vulnerable to SAH induced acute brain injury, which contributes to explain the early, cerebral perfusion pressure-independent severe reduction in cerebral blood flow after SAH. And these findings also provided support for the emerging concept of capillaries, rather than the large vessels, playing a major role in vasospasm induced hypoxic-ischemic impairment^18^,^24^. However, a recent study stated that capillary pericytes, which do not express SMA and have a noncircumferential, longitudinal morphology spanning long distances, do not have the capacity for direct regulation of blood flow^25^. But their experiments was under brain ischemia condition, which was not exact the same as the pearl-like constriction after SAH. Further studies are still needed to verify this phenomenon in the pathophysiology of SAH.

Hemoglobin, which is known to induce vasoconstriction by scavenging NO, was present to be involved in the mechanisms of post hemorrhagic microvessel spasm^15^. Our current data demonstrated that hemoglobin diffused into the brain parenchyma and contacted with microvessel pericyte after SAH, then cause microvessel constriction through NO scavenging in the very short time^15^,^16^, while reduce eNOS expression later to sustain the effect^17^. Furthermore, experimental in brain slices actually showed the hemoglobin could cause microvessel contraction at pericyte sites and block the flow of erythrocytes. Taken together, hemoglobin, which comes from the rupture of erythrocytes, may trigger the pericyte-mediated acute microvessel spasm, which is an emerging recognized risk factor for poor outcome after SAH^18^.

However, due to the inhomogeneously distribution of hemoglobin after initial bleeding and the lack of strategy to clean up hemoglobin inside the parenchyma, it is hard to explore the direct correlation between the presence of hemoglobin and the increase of α-SMA *in vivo*. Our *in vivo* study demonstrated that the distributed hemoglobin in parenchyma could inhibit eNOS/NO signal, and modulating eNOS signal could affect the expression of α-SMA surround microvessel, respectively. Furthermore, our *in vitro* study demonstrated that hemoglobin could inhibit eNOS/NO signal and directly increase the expression of α-SMA in cultured pericyte, which consistent with the results of the *in vivo* study. Taking together, we believed that the increasing of α-SMA expression in brain pericyte is correlated with the presence of hemoglobin in the parenchyma, and further studies are still needed to verify this phenomenon.

In the present study, we found marked decreasing of the eNOS activity and NO availability in brains of SAH model in the early phage, whereas the effects of eNOS expression and/or activity after SAH are still controversial^6^,^26^. We also demonstrated that hemoglobin decreased NO level *in vitro*. Both results are consistent with previous studies in the setting of cerebral ischemia^27^. After SAH, hemoglobin gradually released from blood clot to envelop the conductive arteries, scavenge NO and destroy nNOS-containing neurons. This deprives the cerebral vessels of NO, leading to vasoconstriction which might initiate vasospasm^17^,^28^. Under normal condition, the vessel narrowing increases shear stress and then stimulates eNOS, which would increase the NO availability and dilate those vessels. However, this phenomenon does not occur after SAH, due to the transient eNOS dysfunction evoked by increasing of an endogenous NOS inhibitor, asymmetric dimethylarginine (ADMA)^29^. Increased ADMA levels result from inhibition of the ADMA-hydrolyzing enzyme in constricted vessels by hemoglobin metabolites, bilirubin-oxidized fragments^29^. This eNOS dysfunction sustains vessel constriction until ADMA levels decrease and NO release from endothelial cells increases^17^,^29^.

To verify the effect of NO/cGMP signal in the predicted constriction of pericytes, our data indicated the role of NO on the inducing α-SMA expression, which indicate and regulate pericyte contraction. In addition, Zhang J, *et al.* reported a DCC-ERK1/2-eNOS-NO feed forward loop in cardioprotective effects after ischemic/perfusion injury^30^. Briefly, once the receptor DCC is activated, which will result in ERK1/2/eNOS activation, and then produce NO to upregulate DCC expression, form a feed-forward loop to maintain DCC activity, eNOS expression and additional NO production. These results are similar to previous studies examining NO-induced vasodilation of descending vasa recta^31^, and in the setting of cerebral ischemia^32^. These findings suggested that suppression of NO/cGMP pathway enhances the α-SMA expression in pericyte.

Although the main source of intense NO after brain ischemia/reperfusion injury are the microvessels^32^, eNOS still mostly came from endothelium and nerve ending. However, Zakoji N, *et al.* and Loesch A, *et al.* demonstrated that pericyte could also express eNOS^33^,^34^, which could be one of the reasons that pericyte generates NO under inflammatory condition^35^,^36^. In addition to ipsilateral cortex homogenates, we analyzed the expression and activity of eNOS in cultured pericytes as we showed in Fig. 1E–H. We also found that the same trend as ipsilateral cortex homogenates, which could demonstrate the eNOS is partially came from pericyte and changed among groups in the present study. And it was also reported that pericyte could express iNOS^37^ and nNOS^38^.

However, some limitations of our study should be further studied. Firstly, we used an open cranial window preparation for the current *in-vivo* experiments, which is equivalent to a decompression craniotomy. Accordingly, we cannot make any statements on the role of intracranial hypertension in the observed microcirculatory changes after SAH. Future experiments using closed cranial window preparations will be needed to address this important issue in detail. Secondly, vasospasm in early brain injury occurs mainly within a few hours after the SAH. So, only acute phage reactions were investigated after subarachnoid hemorrhage, but future studies should include a longer observation window. Thirdly, although microvessel constriction is a major contributor to early brain injury, other factors may also play pivotal role in the pathophysiology after SAH. Whether pericytes impairment also affects secondary brain injury, such as blood brain barrier disruption after SAH, should by further studied.

In conclusion, pericyte α-SMA phenotype mediate acute microvessel constriction after SAH possibly by hemoglobin induced suppression of NO/cGMP signaling pathway. By targeting the eNOS and pericyte α-SMA phenotype, our present data and further translational study may shed new light on the management of SAH patients.

## Methods

### Experimental Animals

All experimental protocols were approved by the Ethic Committee of Southwest Hospital, and performed in accordance with the guidelines by the National Institutes of Health Guide for the Care and Use of Laboratory Animals.

One hundred sixty-seven (167) adult six-week-old male Sprague-Dawley rats, weighing 250–320 g and 75 neonatal Sprague-Dawley rats (Animal Center of Daping Hospital, Chongqing, China) were used in the present study. Rats were housed in a humidity and temperature-controlled room with food and water ad libitum. The light was controlled in a 12-hour light/dark cycle. And these rats were acclimatized for more than 3 days before surgical procedures.

### Experimental Design

The experiment was designed as follows (Supplemental Fig. 5).

### Experiment I

To clarify the possible role of eNOS in regulating microcirculation after SAH, 155 rats were randomly assigned into four groups, including Sham (n = 34), SAH + Vehicle (n = 40), SAH + L-NNA (n = 42), and SAH + Scutellarin (n = 41) groups. Modified Garcia test was used to evaluate the neurological deficits at 6, 12, 24 hours after SAH (n = 12). *In vivo* microvessel assessment was used at 3 hours after SAH to evaluate the microvessel condition (n = 8 or 10 depending on the constricted branches arterioles of ipsilateral middle cerebral artery). In addition, western blot at 3 hours after SAH (n = 3), eNOS activity assay and NO availability assay at 3, 6, 12 hours after SAH were also performed to explore the changes of eNOS system (n = 4 per each time point). Furthermore, immunofluorescence staining was used to identify the expression of α-SMA at 12 hours after SAH and the spatial expression of pericyte in both sham and SAH rats (n = 3 per group).

### Experiment II

To explore the potential role of hemoglobin in regulating microcirculation, immunohistochemistry staining of brain tissues and *in vitro* brain slices were used in section. Twelve (12) rats were used to perform the following morphologic assessment. Hematoxylin and eosin staining, hemoglobin α staining by immunohistochemistry and immunofluorescence were used to explore the spatial expression of hemoglobin at 12 hours after SAH (n = 3).

Then, eighteen (18) neonatal rats (P14–P21) were used to harvest *in vitro* brain slices, which were further assigned randomly to be treated with following procedures: 1) control-hemoglobin-washout; 2) control-hemoglobin-DETA/NO; 3) control-L-NNA; 4) control-PTIO. And then, the changes of microvessels were recorded for further analysis for 30 minutes.

In addition, nine (9) neonatal rats (P14–P21) were used to harvest *in vitro* brain slices, which were further assigned randomly to investigate the NO availability and eNOS expressions at 0, 10, 30 minutes and 1, 2 hours after hemoglobin incubation.

### Experiment III

To explain the potential involvement of NO/cGMP signaling pathway in pericyte phenotype transformation, 36 neonatal rats (P1–P3) were used to harvest primary pericytes for *in vitro* culture. Cultured pericytes were randomly assigned to four groups, including Control, Hemoglobin, Hemoglobin + PTIO, and Hemoglobin + DETA/NO groups, then assessed the activity or expression of eNOS and the content of NO in cultured pericyte at 3, 6, 12 hours of incubation. Also, the content of NO in culture medium at 3 hours of incubation, the expressions of α-SMA by western blot and reverse transcription-polymerase chain reaction analyses at 0, 3, 6, 12 hours of incubation was evaluated. Then, another batch of cultured pericytes treated with four dosages (0, 0.1, 1, and 10 mM) of DETA/NO or PTIO plus four dosages (0, 0.1, 1, and 10 mM) of 8-Br-cGMP, and the expressions of α-SMA at 3 hours after 8-Br-cGMP incubation were evaluated by using western blot and reverse transcription-polymerase chain reaction.

To double check the biological relevance of NO/cGMP signal, twelve (12) neonatal rats (P14–P21) were used to harvest *in vitro* brain slices, which were further assigned randomly to four groups, including Control, Hemoglobin, Hemoglobin + PTIO, and Hemoglobin + DETA/NO groups, then assessed content of NO and the expressions of α-SMA at 3 hours of incubation, as well as the time course of α-SMA expressions at 0, 10, 30 minutes and 1, 2 hours of solo hemoglobin incubation. Moreover, the expressions of α-SMA at 3 hours after PTIO with/without 8-Br-cGMP incubation were evaluated by using western blot.

### Endovascular Perforation Model of SAH and Drug Administration

The endovascular perforation model of SAH was performed as described previously^39^. The details were described in the Supplementary Materials.

Buprenorphine was used depending on the degree of observed distress or pain, and given for 6 hours-1 day depending on signs of pain or distress. The first administration of buprenorphine (0.02 mg/kg) was injected subcutaneously before anesthetic recovery and rats were observed for any sign of pain or distress.

Nω-Nitro-L-arginine (L-NNA), a pan-NOS inhibitor, was intraperitoneally injected at the dosage of 10 mg/kg^40^ right after SAH. Scutellarin, an eNOS agonist, was also intraperitonieally injected at the dosage of 50 mg/kg^41^ at the time of SAH. Sterile saline was used as vehicle in the present study.

### Modified Garcia Test

As previous study^42^, an 18-point scoring system was used to evaluate six aspects of neurologic deficits in animals. The details were described in the Supplementary Materials.

### *In Vivo* Microvessel Assessment

Intravital fluorescence microscopy was performed at 3 hour after SAH for microvessel assessment as previously described^43^,^44^. Briefly, animals were anesthetized and a cranial window (4 × 4 mm) was drilled above the left hemisphere (Supplemental Fig. 1A). The microcirculation was visualized by FITC-dextran (0.5%, 10 mg/kg, Sigma-Aldrich, St. Louis, MO) intravenous injection into left femoral vein. Entire vascular tree of the ipsilateral middle cerebral artery (vessel diameter: 5 to 20 μm; 10 to 16 vessel segments per rat) was visualized in each rat. Each segment, which possibly containing more than one constricted section, was investigated. Images were captured by a CCD camera and analyzed offline by a blinded investigator.

Microvessel diameter was assessed by using the methods of Friedrich B, *et al.*^43^ with slightly modification. Briefly, vessel diameters were quantified in the images we captured by a CCD camera, and analyzed by using Imaging J software (National Institutes of Health, Bethesda, MD) as previously described^44^,^45^. Individual vessel constrictions were analyzed by dividing the diameter of the most constricted vessel segment by the diameter of the nearest not constricted vessel segment. To validate that not constricted appearing vessel segments indeed represented the baseline diameter of this vessel, the mean diameter of each category was calculated and compared between sham-operated rats and SAH rats. No difference was detected between groups indicating that nonconstricted vessel segments in mice subjected to SAH indeed represented the physiological baseline.

### Post-Surgical Monitoring and Sacrificing

After surgical procedures, rats were housed separately in clear cages with free access to rat chow and filtered water. And surgeons will check their general condition at least four times per day. At the designed time as experimental protocol, rats were anesthetized with 5% chloral hydrate (350 mg/kg, intraperitoneally), and then were decapitated by guillotine. Brain specimen were removed carefully and prepared for the following experiments.

### SAH Grade

All rats received an 18-point SAH severity grading after sacrificing as previous^46^. The details were described in the Supplementary Materials.

### eNOS Activity Assay

eNOS activity was measured by the conversion of l-arginine to NO by using Nitric-Oxide Synthase Assay Kit (Nanjing Jiancheng Bioengineering Institute, Nanjing, China). The details were described in the Supplementary Materials.

### NO Availability Assay

Nitric oxide content in the medium and brain tissue was measured by using NO Detection Kit (Nanjing Jiancheng Biotechnology, Nanjing, China) as previously described^47^. The details were described in the Supplementary Materials.

### Hematoxylin and Eosin Staining, Immunohistochemistry Staining and Immunofluorescence Staining

Hematoxylin and eosin staining and following immunohistochemistry and immunofluorescence staining were performed as described previously^47^. Primary antibody: anti-Hbα (1: 200, Abcam, Cambridge, United Kingdom), anti-α-SMA (1:200 Santa Cruz Biotechnology, Santa Cruz, CA), anti-PDGFRβ (1:100, Abcam, Cambridge, United Kingdom), anti-Hbα and anti-CD34 (1:200, Abcam, Cambridge, United Kingdom), anti-IB4 (1:150, Invitrogen, Shanghai, China), anti-NG2(1:150, Thermo Scientific Pierce, Shanghai, China). The details were described in the Supplementary Materials.

### Microvessel Imaging in Brain Slices

Brain slices (300 mm thick) were prepared, as previously reported^47^, on vibratome in ice-cold oxygenated (95%O_2_, 5%CO_2_) artificial cerebrospinal fluid solution as previously reported^7^, which contains 124 mM NaCl, 2.5 mM KCl, 26 mM NaHCO_3_, 1 mM MgCl_2_, 1 mM NaH_2_PO_4_, 10 mM glucose, 0.1–1 mM Na-ascorbate, and 2 mM CaCl_2_. Those slices were incubated at room temperature in the same solution until using. Then, brain slices were perfused with hemoglobin (10 μM), hemoglobin (10 μM) +DETA/NO (10 μM), L-NNA (25 μM), and PTIO (10 μM), respectively. For bright-field recording of microvessel diameter, sagittal cerebellar slices were prepared from neonatal (P14–P21) rats. On each slice, microvessels were imaged at approximately 30 mm depth by using a ×40 water immersion objective, a Coolsnap HQ2 CCD camera (Princeton Instruments, Buckinghamshire, United Kingdom), and ImagePro Plus software. Because this depth is within the molecular layer of cerebellar slices, or the grey matter of slices containing the somatosensory and motor cortices. Images were acquired every 30 seconds, and 20–30 minutes in total after perfusion was investigated. Vessel internal diameters were measured by manually placing a measurement line on the image using Image Pro Analyzer software.

### Brain Pericyte Culture

Pericyte culture was performed as previously reported^48^,^49^. The details were described in the Supplementary Materials.

### Western Blot

Western blots were performed as described previously^47^. The following primary antibodies were used: anti-α-SMA (1:1000, Abcam, Cambridge, United Kingdom), anti-eNOS (1:2000, Abcam, Cambridge, United Kingdom), and anti-GAPDH (1:2000, DAKO, Glostrup, Denmark). The details were described in the Supplementary Materials.

### Reverse Transcription-Polymerase Chain Reaction

Reverse Transcription-Polymerase Chain Reaction was performed as previously described^50^. The details were described in the Supplementary Materials. Primer sequences were described as follow: α-SMA forward 5′-CTGGCATCGTGCTGGACTC-3′ reverse 5′-GCCCATCAGGCAACTCGTA-3′ (291 bp) GAPDH forward 5-TGATGACATCAAGAAGGTGGTGAA-3′ reverse 5′-TCCTTGGAGGCCATGTGGGCCAT-3′ (256 bp).

### Statistics

All data were presented as mean ± standard deviation and analyzed by SPSS 13.0 or GraphPad Prism 5 software. Chi-square test was used for the statistical analysis of neurological deficits between different time and groups. For statistical analysis of microvessels, independent samples t-test was used. For time course of NO and eNOS after hemoglobin incubation in cultured brain slices, Student’s t test was used. Rest data were assessed by one-way analysis of variance followed by Bonferroni multiple comparison method. A probability level of p < 0.05 was considered statistically significant.

## Additional Information

**How to cite this article**: Li, Q. *et al.* Hemoglobin induced NO/cGMP suppression Deteriorate Microcirculation via Pericyte Phenotype Transformation after Subarachnoid Hemorrhage in Rats. *Sci. Rep.* **6**, 22070; doi: 10.1038/srep22070 (2016).

## Supplementary Material

Supplementary Information
