# Author Correction: Hemoglobin induced NO/cGMP suppression Deteriorate Microcirculation via Pericyte Phenotype Transformation after Subarachnoid Hemorrhage in Rats

**DOI:** 10.1038/s41598-024-64285-0

**Published:** 2024-06-10

**Authors:** Qiang Li, Yujie Chen, Bo Li, Chunxia Luo, Shilun Zuo, Xin Liu, John H. Zhang, Huaizhen Ruan, Hua Feng

**Affiliations:** 1 Department of Neurosurgery, Southwest Hospital, Third Military Medical University, Chongqing, China; 2 Department of Neurobiology, College of Basic Medical Sciences, Third Military Medical University, Chongqing, China; 3 Department of Neurosurgery, Jinan Military General Hospital, Jinan, Shandong China; 4 Department of Neurology, Southwest Hospital, Third Military Medical University, Chongqing, China; 5 Department of Physiology and Pharmacology, Loma Linda University, Loma Linda, CA USA

Correction to: *Scientific Reports* 10.1038/srep22070, published online 25 February 2016

This Article contains an error in Figure 5B, in the 3rd and 4th panels. The images were found to be partially overlapped, and upon further review of the raw data and experimental recordings, it is confirmed that both images belong to the same experimental group, SAH + Scutellarin. This error does not affect the overall conclusions of the study.

The corrected Figure 5 and accompanying legend appear below.

**Figure 5:**
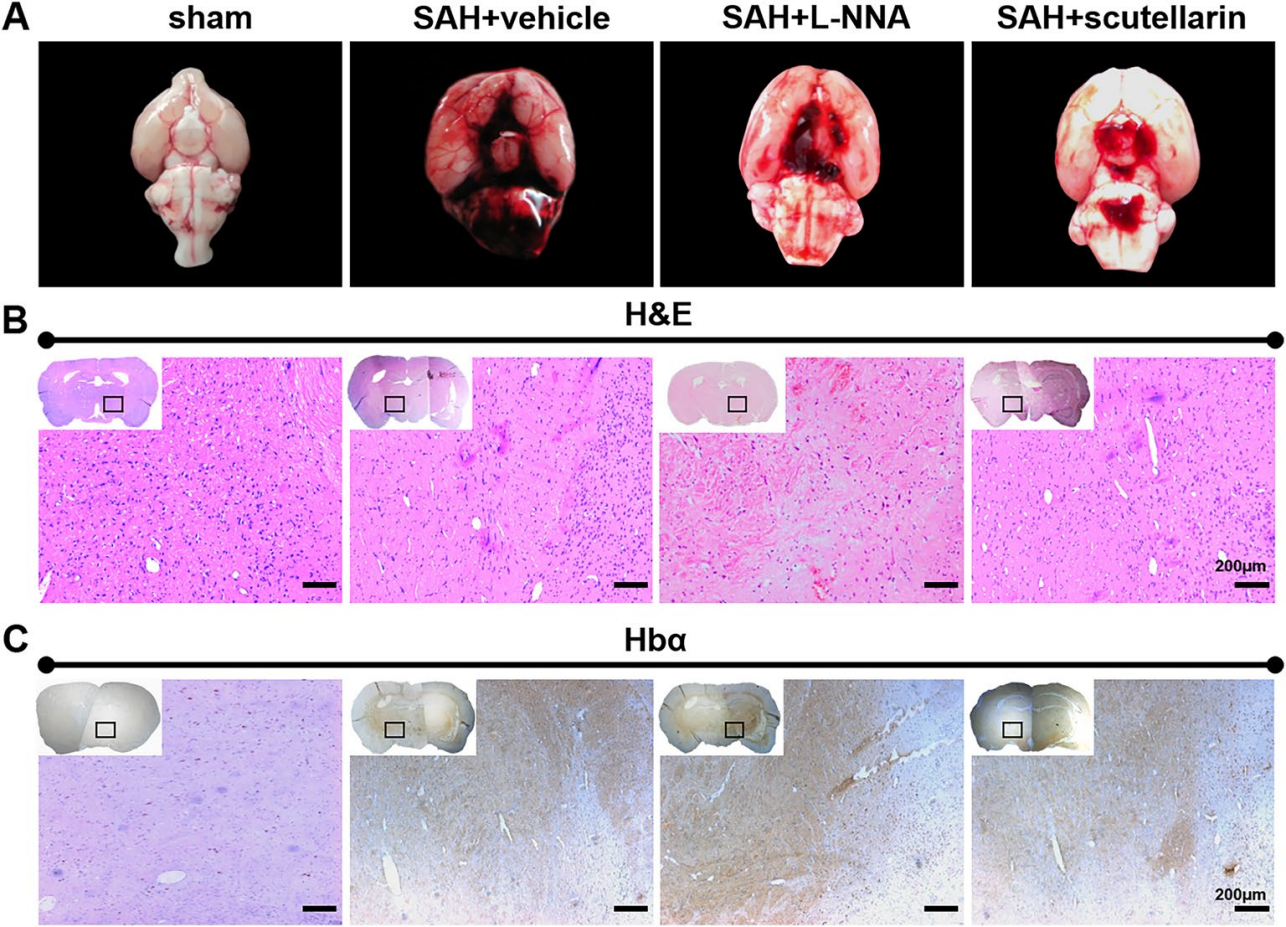
Distribution of leaked blood and hemoglobin at 12 h after SAH. (**A**) Representative pictures of the skull base after SAH surgery. (**B**) Representative pictures of hematoxylin and eosin staining for blood distribution after SAH. (**C**) Representative pictures of immunohistochemistry staining for hemoglobin-α. Hbα: hemoglobin-α; Scale bars = 200 μm; n = 3 in each group.

