# Supplementary material for: Hemoglobin induced NO/cGMP suppression Deteriorate Microcirculation via Pericyte Phenotype Transformation after Subarachnoid Hemorrhage in Rats
Source: Sci Rep. 2016 Feb 25;6:22070. doi: 10.1038/srep22070 (PMC4766506; doi:10.1038/srep22070)
Supplement: Supplementary Information [file srep22070-s1.pdf]

Hemoglobin induced NO/cGMP suppression Deteriorate Microcirculation via Pericyte Phenotype Transformation after Subarachnoid Hemorrhage in Rats

Qiang Li[1, 2, #]; Yujie Chen[1, #]; Bo Li[3]; Chunxia Luo[4]; Shilun Zuo[1]; Xin Liu[1]; John H. Zhang[4]; Huaizhen Ruan[2, *]; Hua Feng[1, *]

1. Department of Neurosurgery, Southwest Hospital, Third Military Medical University, Chongqing, China

2. Department of Neurobiology, College of Basic Medical Sciences, Third Military Medical University, Chongqing, China

3. Department of Neurosurgery, Jinan Military General Hospital, Jinan, Shandong, China

4. Department of Neurology, Southwest Hospital, Third Military Medical University, Chongqing, China

5. Department of Physiology and Pharmacology, Loma Linda University, Loma Linda, California, USA

# Both authors contributed equally to this work

Correspondense to:

Hua Feng, MD, PhD

Department of Neurosurgery, Southwest Hospital, Third Military Medical University. 29 Gaotanyan Street, Shapingba District, Chongqing, 400038, China

Fax: 86-23-68754153

Tel: 86-13708321681

Email: fenghua8888@vip.163.com

Huaizhen Ruan, PhD

Department of Neurobiology, College of Basic Medical Sciences, Third Military Medical University. 30 Gaotanyan Street, Shapingba District, Chongqing, 400038, China

Fax: 86-23-68754153

Tel: 86-13228686299

Email: hzruan61@yahoo.com

Supplemental Figures

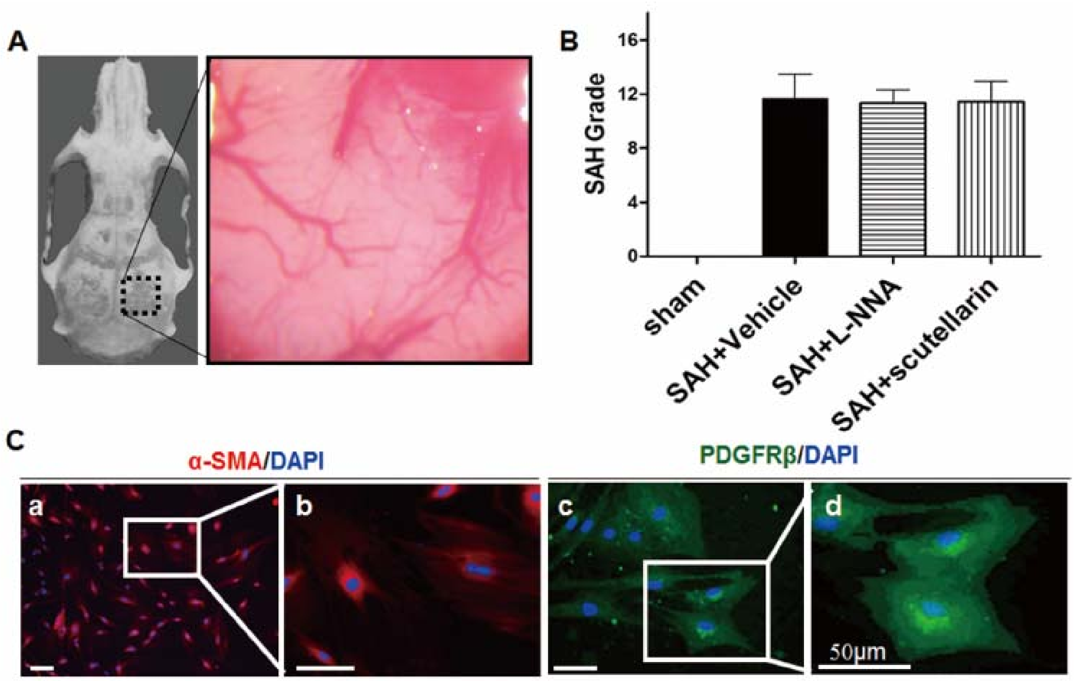

Supplemental Figure 1. Cranial window, SAH grade, and cultured pericytes in the present study.

(A) Schematic drawing of the location of the cranial window used for intravital microscopy. (B) SAH grade of rats in each group; (C) Representative immunofluorescence staining pictures of the cultured pericytes.

Scale bar=25μm. All data were presented as mean ± standard deviation. Data were analyzed by one-way analysis of variance followed by Newman-Keuls multiple comparison method.

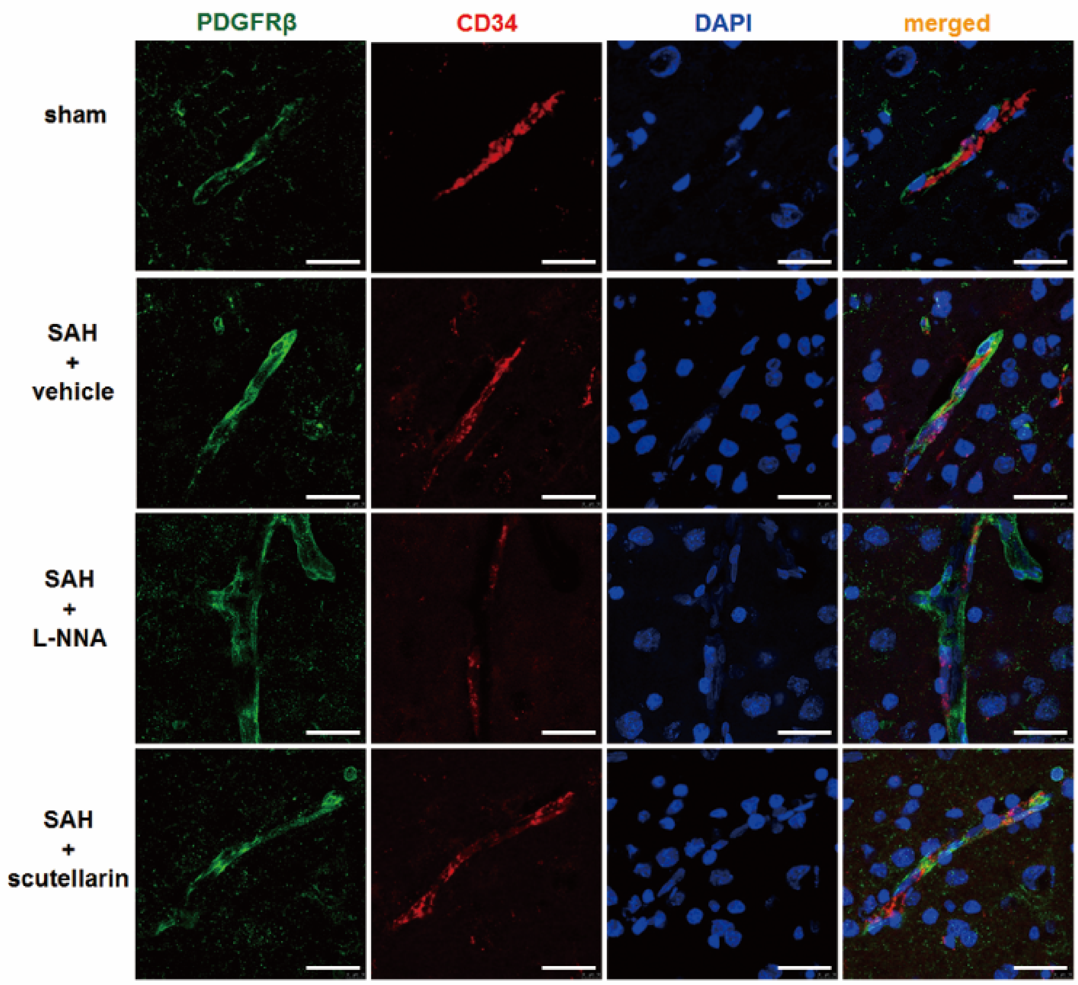

Supplemental Figure 2. The spatial expression of pericytes.

Representative double immunofluorescence pictures of the co-localization of PDGFRβ and CD34 at 12 hours after SAH.

PDGFRβ: platelet-derived growth factor receptor beta; Scale Bar=20μm; n=3 in each group.

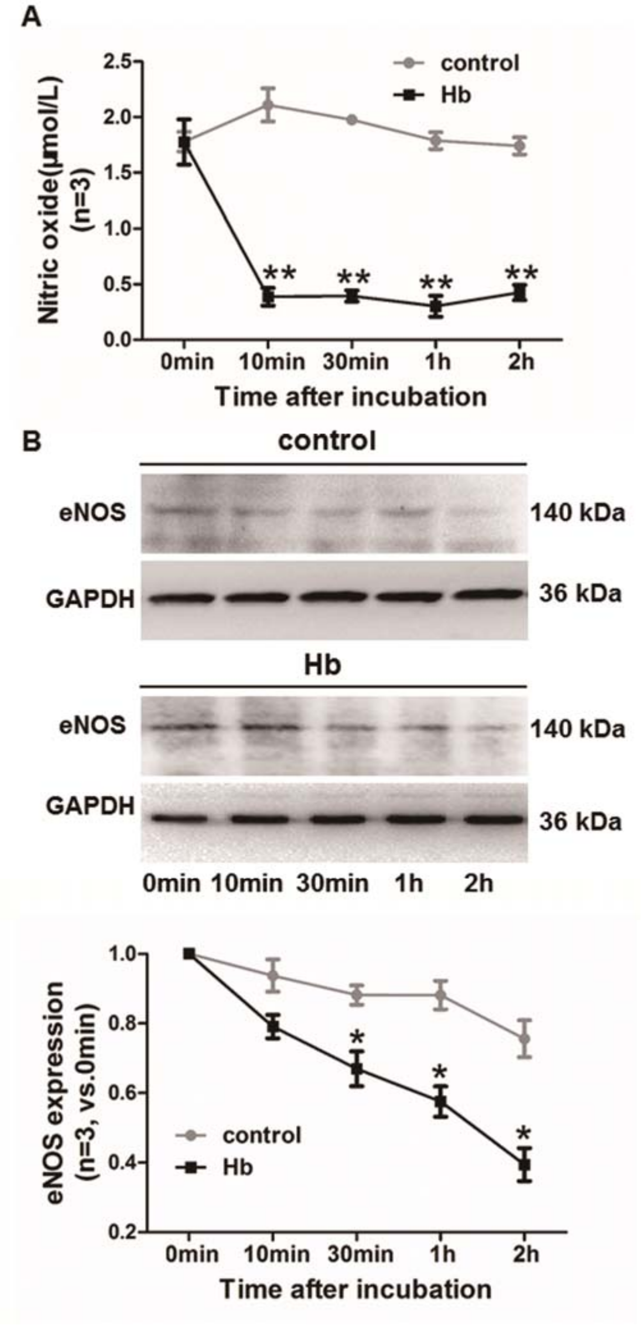

Supplemental Figure 3. The time course of NO and eNOS after hemoglobin incubation in cultured brain slices.

(A) The NO availability at 0, 10, 30 minutes and 1, 2 hours after hemoglobin incubation in cultured brain slices. (B) Representative bands and quantitative

analysis of eNOS expressions at 0, 10, 30 minutes and 1, 2 hours after hemoglobin incubation in cultured brain slices. The cropped bands had been run under the same experimental conditions.

NO: nitric oxide; eNOS: endothelial nitric oxide synthase. All data were presented as mean ± standard deviation. Data were analyzed by Student's t test. * $p < 0.05$ vs. Control; ** $p < 0.01$ vs. Control; n=3 in each group.

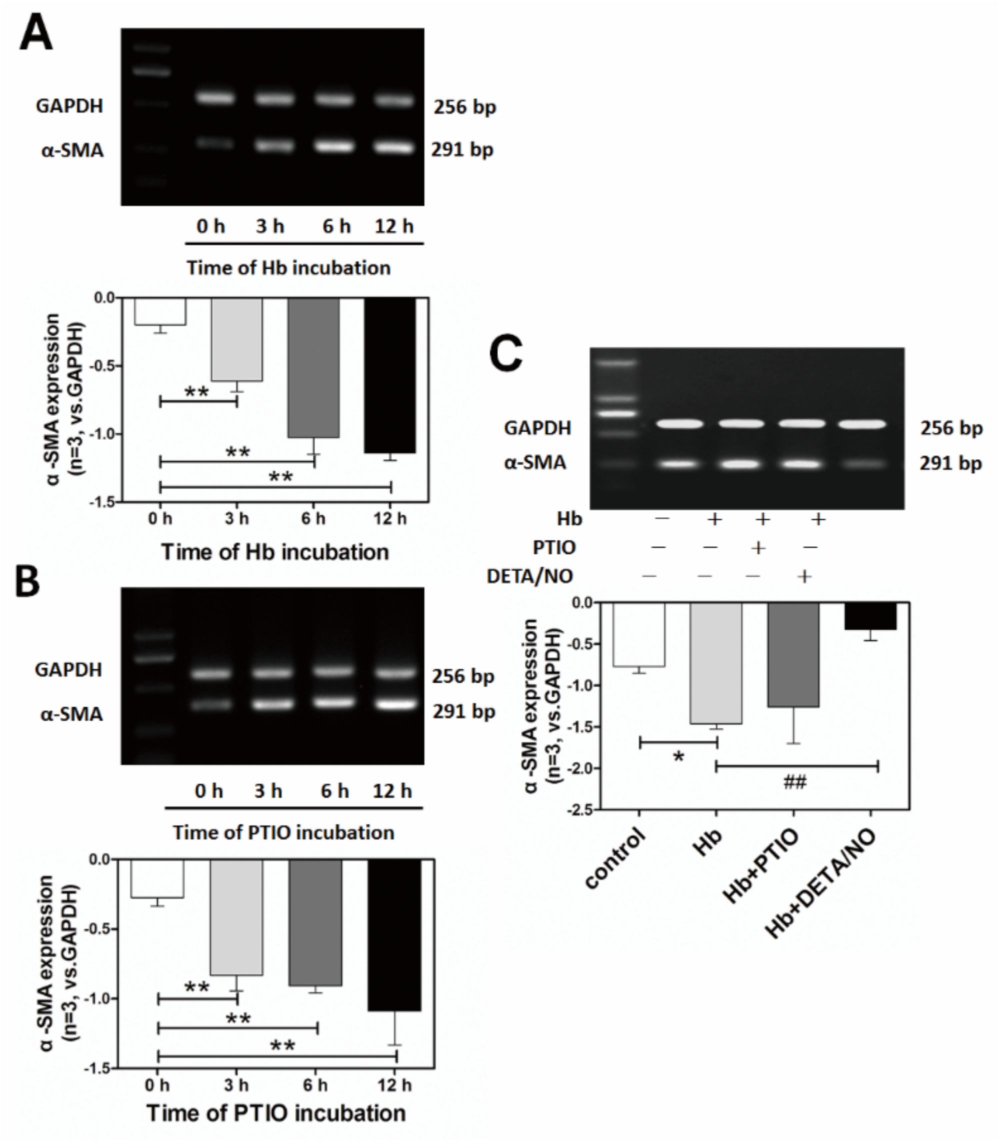

Supplemental Figure 4. Effects of nitric oxide/cGMP signal on the miRNA levels of α-SMA in cultured pericytes.

(A) Representative RT-PCR bands and quantification analysis of α-SMA at 3, 6, and 12 hours after hemoglobin incubation. (B) Representative RT-PCR bands and quantification analysis of α-SMA at 3, 6, and 12 hours after PTIO incubation. (D) Representative RT-PCR bands and quantification analysis of α-SMA after 3 hours incubation in each group. The cropped bands had been run under the same experimental conditions.

RT-PCR: reverse transcription-polymerase chain reaction. All data were presented as mean ± standard deviation. Data were analyzed by one-way analysis of variance followed by Newman-Keuls multiple comparison method. * $p < 0.05$ vs. Control or zero hours after incubation; ** $p < 0.01$ vs. Control or zero hours after incubation; ## $p < 0.01$ vs. Hemoglobin; n=3 in each group.

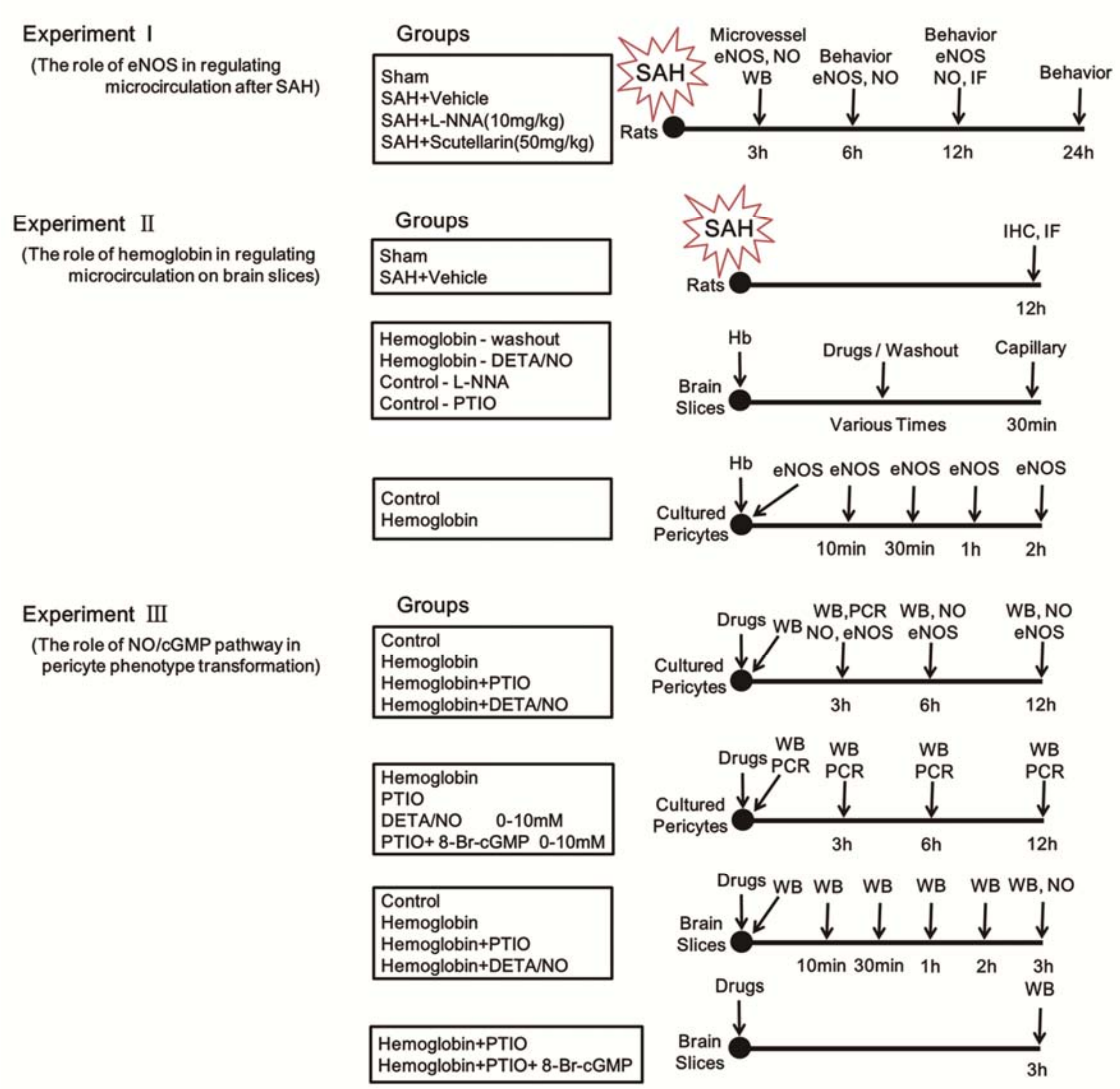

Supplemental Figure 5. Experimental design and groups' classification.

SAH: subarachnoid hemorrhage; NO: nitric oxide; eNOS: endothelial nitric oxide synthase; Capillary: capillary assessment in vivo or in vitro brain slices; WB: western blot; IHC: immunohistochemistry staining; IF: immunofluorescence; Behavior: neurobehavioral test; Hb: hemoglobin; PCR: reverse transcription-polymerase chain reaction.

Supplemental Information of Methods

Endovascular Perforation Model of SAH

The endovascular perforation model of SAH was performed as described previously[1]. Briefly, under 5% chloral hydrate (350mg/kg, intraperitoneally) anesthesia, a sharpened 4–0 monofilament nylon suture was advanced rostrally into the right internal carotid artery from the external carotid artery stump to perforate the bifurcation of the right anterior and middle cerebral arteries. Rectal temperature was kept at 37°C during surgical procedure with an electronic blanket. Sham-operated rats underwent identical procedures without perforation.

Modified Garcia Test

As previous study[2], an 18-point scoring system was used to evaluate six aspects of neurologic deficits in animals, including spontaneous activity, symmetry in the movement of all four limbs, forepaw outstretching, climbing, body proprioception, and response to vibrissae touch. Each subtest was scored between 0 to 3 points. Lower scores indicated more neurobehavioral deficits. This test was performed in a blinded manner to avoid bias.

SAH Grade

All rats received an 18-point SAH severity grading after sacrificing as previous described[3]. Briefly, basal cistern was divided into 6 segments and scored from 0 to 3 point by a blinded observer according to the amount of subarachnoid

blood. A total score ranged from 0 to 18 representing the severity of the SAH. Rats received a score less than 8 were excluded for the following studies.

eNOS Activity Assay

eNOS activity was measured by the conversion of l-arginine to NO by using Nitric-Oxide Synthase Assay Kit (Nanjing Jiancheng Bioengineering Institute, Nanjing, China). One unit of NOS activity was defined as the production of 1 nmol nitric oxide per second per microgram tissue protein. Total NOS activity was measured as follows: 10% tissue homogenate (100 μl) was incubated with 200 μl substrate buffer, 10 μl reaction accelerator and 100 μl color development reagent at 37 ℃ for 15 minutes after mixing. Then 100 μl clearing reagent and 2 mL stop solution were added and mixed. Then, the absorbance was read at 530 nm. For measuring eNOS activity, an inhibitor was added before incubation according to the manufacturer's instructions. The results were expressed as U/mg.

NO Availability Assay

Nitric oxide content in the medium and brain tissue was measured by using NO Detection Kit (Nanjing Jiancheng Biotechnology, Nanjing, China) as previously described[4]. Briefly, the tissue solution was collected and assayed in triplicate, and a standard curve using $NaNO_2$ was generated for each experiment for quantification. Then, 50μl of the supernatant or $NaNO_2$ standard was mixed with 50μl of Griess reagent I and 50μl of Griess reagent II in a 96-well plate at room

temperature for 10 minuntes, and the absorbance was measured at 540 nm using a microplate reader. The results were expressed as μg/mL.

Hematoxylin and Eosin Staining

Hematoxylin and eosin staining and following immunohistochemistry and immunofluorescence staining were performed as described previously[4]. Briefly, rats were anesthetized and perfused through the ascending aorta with sterile saline, followed by 4% paraformaldehyde in phosphate buffer (0.1M sodium phosphate, pH 7.4). Brain specimen were removed, post-fixed for 24 hours in phosphate-buffered 4% paraformaldehyde, embedded with paraffin, and cut into 6μm sections on a vibratome. Paraffin sections were deparaffinizated in xylene and rehydrated in a decreasing gradient of ethanol. Then, slides were stained with hematoxylin and eosin. After dehydration in an increasing gradient of ethanol and three times of xylene, slides were covered and viewed under a light microscope.

Immunohistochemistry Staining

Paraffin sections were prepared as hematoxylin and eosin staining and treated with 0.3% Triton and 3% H_2O_2, then incubated for 24 hours at 4°C with anti-Hbα primary antibody (1: 200, Abcam, Cambridge, United Kingdom) in 2% goat serum. After three time washes, the sections were treated according to the instructions of the Streptavidin-Peroxidase Kit (Zhongshan Goldenbridge Biotechnology, Beijing, China). After that, those sections were incubated with

0.6mg/ml diaminobenzidine and 0.05% H_2O_2 for 2 minutes. Then the sections were coverslipped onto gelatin-coated slides, and observed under a light microscope.

Immunofluorescence Staining

Brain specimen was prepared as hematoxylin and eosin staining, and embedded in OCT and cut into 10μm coronal sections. Cryosection sections were soaked in acetone for 1 minute at room temperature, and then rehydrated in phosphate buffer solution, blocked in 5% goat serum, stained with the anti-α-SMA (1:200 Santa Cruz Biotechnology, Santa Cruz, CA), anti-PDGFRβ (1:100, Abcam, Cambridge, United Kingdom), anti-Hbα and anti-CD34 (1:200, Abcam, Cambridge, United Kingdom), anti-IB4 (1:150, Invitrogen, Shanghai, China), anti-NG2(1:150, Thermo Scientific Pierce, Shanghai, China) followed by appropriate Alexa-fluorophore-conjugated secondary antibodies (Abcam, Cambridge, United Kingdom). Then, all sections were incubated with 4, 6-diamidino-2-phenylindole for 10 minutes. Slides were mounted in antifade regent (Beyotime, Guangzhou, China) and visualized by a fluorescence microscopy. The fluorescence intensity was analyzed by using Image J software in a blinded manner.

Brain Pericyte Culture

Pericyte culture was performed as previously reported[5,6]. Brains were harvest from 6 rats (P1-P3) and digested in an enzymatic solution, which contains 30U/mL

papain and 40μg/ML DNAse I in HEPES Balanced Salt Solution. Then, digestion was terminated in phosphate buffered saline with 1.7 volumes of 22% bovine serum albumin, and followed by centrifuging at 4000 rpm for 10 minutes in Eppendorf 5810R Centrifuge with a 4-62 rotor (swing-bucket rotor with 4 x 250 ml rectangular buckets). Cells in the lower layer were re-suspended in endothelial cell growth medium consisting of Hams F12, supplemented with 10% fetal bovine serum, heparin, ascorbic acid, L-glutamine, penicillin/streptomycin and endothelial cell growth supplement (ScienCell Research Laboratories, San Diego, CA). Then these cells was plated in a six-well plate coated with collagen I (0.02%, Sigma-Aldrich, St. Louis, MO) for 2 hours at 37 °C. The first passage was performed at the 7–9th day. After that, cells were culture in pericyte medium (ScienCell Research Laboratories, San Diego, CA) containing 2% fetal bovine serum, and could be used after the third passage. To be identified with immunocytochemical analysis, cells were treated under the same conditions as described earlier but were passaged onto collagen-coated slides and grown for 3 days unless indicated.

Western Blot

Western blots were performed as described previously[4]. Briefly, protein from ipsilateral cortex or cultured pericytes were harvested at designed time as experimental protocols. Samples were lysed and placed on ice for 30 minutes for centrifugation. Total protein was measured by a bicinchoninic acid assay

(Beyotime, Guangzhou, China). The following primary antibodies were used: anti-α-SMA (1:1000, Abcam, Cambridge, United Kingdom), anti-eNOS (1:2000, Abcam, Cambridge, United Kingdom), and anti-GAPDH (1:2000, DAKO， Glostrup, Denmark). Blots were subsequently incubated with relative horseradish peroxidase-conjugated IgG and visualized by using Chemiluminescence Kit (Beyotime, Guangzhou, China). The images were scanned with a GS800 densitometer scanner (Bio-Rad, Hercules, CA), and OD data were analyzed by using Quantity One software. In these analyses, GAPDH was used as an internal reference.

Reverse Transcription-Polymerase Chain Reaction

Reverse Transcription-Polymerase Chain Reaction was performed as previously described[7]. Briefly, total RNA was extracted and purified from pericytes by using an RNeasy Mini Kit (Toyobo, Osaka, Japan), and measured by ultraviolet spectroscopy. cDNA was synthesized from 2μg of total RNA by using Oligo (dT) primer (Invitrogen, Grand Island, NY). The expressions of target mRNAs were detected by reverse transcription polymerase chain reaction system (Toyobo, Osaka, Japan). Polymerase chain reaction products were electrophoresed on a 1.5% agarose gel, and DNA bands were visualized by ethidium bromide. Primer sequences were described as follow:

α-SMA forward 5′-CTGGCATCGTGCTGGACTC-3′

reverse 5′-GCCCATCAGGCAACTCGTA-3′ (291bp)

GAPDH forward 5-TGATGACATCAAGAAGGTGGTGAA-3′

reverse 5′-TCCTTGGAGGCCATGTGGGCCAT-3′ (256bp)

Relative expression of each mRNA was calculated by grey value fold method and analyzed by using Quantity One software.

References

1 Chen, Y. *et al.* Norrin protected blood-brain barrier via frizzled-4/beta-catenin pathway after subarachnoid hemorrhage in rats. *Stroke* **46**, 529-536, doi:10.1161/STROKEAHA.114.007265 (2015).

2 Chen, Y. *et al.* Administration of a PTEN inhibitor BPV(pic) attenuates early brain injury via modulating AMPA receptor subunits after subarachnoid hemorrhage in rats. *Neurosci Lett* **588**, 131-136, doi:10.1016/j.neulet.2015.01.005 (2015).

3 Sugawara, T., Ayer, R., Jadhav, V. & Zhang, J. H. A new grading system evaluating bleeding scale in filament perforation subarachnoid hemorrhage rat model. *J Neurosci Methods* **167**, 327-334, doi:10.1016/j.jneumeth.2007.08.004 (2008).

4 Li, B. *et al.* Role of HCN channels in neuronal hyperexcitability after subarachnoid hemorrhage in rats. *J Neurosci* **32**, 3164-3175, doi:10.1523/JNEUROSCI.5143-11.2012 (2012).

5 Boroujerdi, A., Tigges, U., Welser-Alves, J. V. & Milner, R. Isolation and culture of primary pericytes from mouse brain. *Methods Mol Biol* **1135**, 383-

392, doi:10.1007/978-1-4939-0320-7_31 (2014).

6 Li, Q. *et al.* [Cell culture and identification of pericytes in rat brain]. *Zhonghua Bing Li Xue Za Zhi* **42**, 551-553 (2013).

7 Yang, Y. F. *et al.* Interleukin-1 receptor associated kinases-1/4 inhibition protects against acute hypoxia/ischemia-induced neuronal injury in vivo and in vitro. *Neuroscience* **196**, 25-34, doi:10.1016/j.neuroscience.2011.08.059 (2011).
